# (*E*)-3-(9-Ethyl-9*H*-carbazol-3-yl)-1-(2-meth­oxy­phen­yl)prop-2-en-1-one

**DOI:** 10.1107/S1600536814001263

**Published:** 2014-01-22

**Authors:** Hongshan Lai, Judith C. Gallucci, Chenglong Li

**Affiliations:** a Division of Medicinal Chemistry and Pharmacognosy, College of Pharmacy, The Ohio State University, Columbus, OH 43210, USA; b Department of Chemistry and Biochemistry, 100 West 18th Avenue, The Ohio State University, Columbus, OH 43210, USA

## Abstract

In the title mol­ecule, C_24_H_21_NO_2_, the dihedral angle between the carbazole ring system [with a maximum deviation of 0.052 (2) Å] and the benzene ring is 38.6 (1)°. In the crystal, weak bifurcated (C—H)_2_⋯O hydrogen bonds link the mol­ecules into chains along [100].

## Related literature   

For biological applications of the title compound, see: Caulfield *et al.* (2002 ▶). For the synthesis, see: Mazimba *et al.* (2011 ▶). For a related structure, see: Cao *et al.* (2005 ▶).
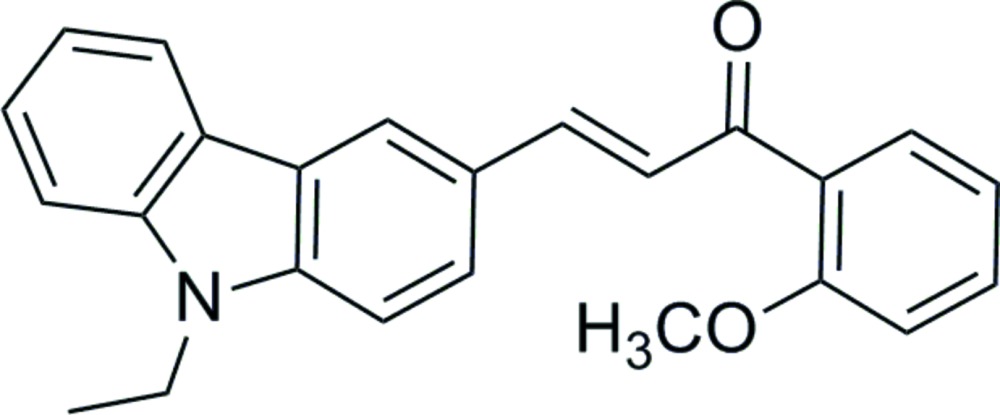


## Experimental   

### 

#### Crystal data  

C_24_H_21_NO_2_
*M* *_r_* = 355.42
Orthorhombic, 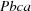

*a* = 15.9332 (7) Å
*b* = 7.8915 (3) Å
*c* = 30.4062 (14) Å
*V* = 3823.2 (3) Å^3^
*Z* = 8
Mo *K*α radiation
μ = 0.08 mm^−1^
*T* = 150 K
0.35 × 0.27 × 0.02 mm

#### Data collection  

Nonius KappaCCD diffractometer
Absorption correction: multi-scan (*HKL* *SCALEPACK*; Otwinowski & Minor, 1997 ▶) *T*_min_ = 0.746, *T*_max_ = 0.998
31757 measured reflections
3352 independent reflections
1845 reflections with *I* > 2σ(*I*)
*R*_int_ = 0.070

#### Refinement  

*R*[*F* ^2^ > 2σ(*F* ^2^)] = 0.048
*wR*(*F* ^2^) = 0.102
*S* = 1.00
3352 reflections
246 parameters
H-atom parameters constrained
Δρ_max_ = 0.16 e Å^−3^
Δρ_min_ = −0.14 e Å^−3^

### 

Data collection: *COLLECT* (Bruker, 2008 ▶); cell refinement: *HKL* *SCALEPACK* (Otwinowski & Minor 1997 ▶); data reduction: *HKL* *DENZO* (Otwinowski & Minor 1997 ▶) and *SCALEPACK*; program(s) used to solve structure: *SHELXS2013* (Sheldrick, 2008 ▶); program(s) used to refine structure: *SHELXL2013* (Sheldrick, 2008 ▶); molecular graphics: *ORTEP-3 for Windows* (Farrugia, 2012 ▶) and *PLATON* (Spek, 2009 ▶); software used to prepare material for publication: *WinGX* publication routines (Farrugia, 2012 ▶).

## Supplementary Material

Crystal structure: contains datablock(s) global, I. DOI: 10.1107/S1600536814001263/lh5681sup1.cif

Structure factors: contains datablock(s) I. DOI: 10.1107/S1600536814001263/lh5681Isup2.hkl

Supporting information file. DOI: 10.1107/S1600536814001263/lh5681Isup3.cml

CCDC reference:

Additional supporting information:  crystallographic information; 3D view; checkCIF report

## Figures and Tables

**Table 1:** Hydrogen-bond geometry (Å, °)

*D*—H⋯*A*	*D*—H	H⋯*A*	*D*⋯*A*	*D*—H⋯*A*
C2—H2⋯O1^i^	0.95	2.48	3.387 (3)	160
C13—H13*A*⋯O1^i^	0.99	2.47	3.379 (3)	153

Symmetry code: (i) 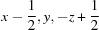.

**Fig. 1.**
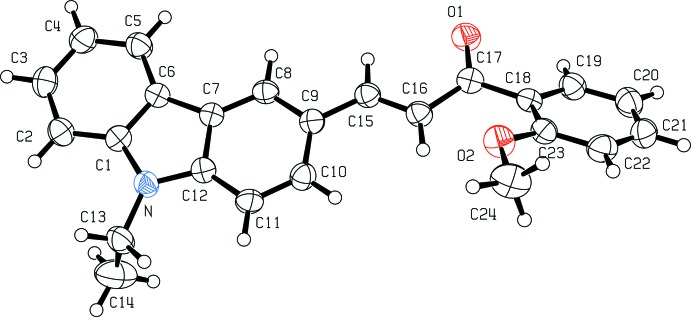
The molecular structure of (I) with displacement ellipsoids drawn at the 50% probability level (arbitrary spheres for the H atoms).

**Fig. 2.**
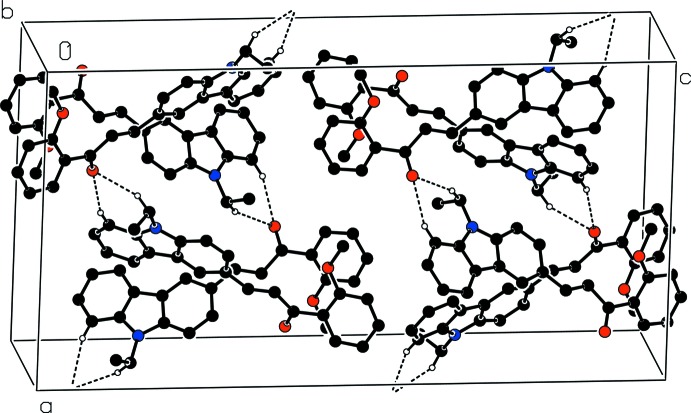
Crystal packing of (I) showing weak C—H···O hydrogen bonds as dashed lines. Only H atoms involved in these hydrogen bonds are shown.

## supplementary crystallographic information

### 1. Comment

The title compound (I) exhibits potential tubulin polymerization-inhibiting activity in drug discovery (Caulfield *et al.*, 2002). It was obtained by reacting 1-(2-methoxyphenyl)ethanone and 9-ethyl-9*H*-carbazole-3-carbaldehyde through a modified procedure from Mazimba *et al.* (2011). The molecular structure of (I) is shown in Fig. 1. The fused ring system and the benzene ring are linked via an α,β-unsaturated carbonyl group so that the molecule has the potential to contain an extended π-conjugation system. However, there is distortion from a planar conformation, which is reflected in the dihedral angle between the carbazole ring system (with a maximum deviation of 0.052 (2)Å for C10) and the benzene ring which is 38.6 (1)°. The α, β-unsaturated carbonyl group is close to planar with a torsion angle of -4.2 (3)° for C15—C16—C17—O1 and forms a dihedral angle of 7.9 (1)° with the carbazole group, indicating some π conjugation between these two groups. A similar structure has been reported where the methoxy group on the benzene ring is substituted by a hydroxyl group (Cao *et al.*, 2005). In this hydroxyl derivative the molecule is more planar, with a dihedral angle of 11.58 (12)° between the planes of the carbazole ring system and benzene ring. Also in the hydroxyl derivative, the hydroxyl group is involved in an intramolecular hydrogen bond with the oxygen atom of the ketone group. In molecule (I), the methoxy group is on the opposite side of the molecule from the ketone group as a result of an approximate 180° rotation about the C17—C18 bond, possibly to avoid steric interactions between atoms O1 and O2. In the crystal, weak bifurcated (C—H)_2_···O hydrogen bonds link molecules into chains along [100] (Fig. 2).

### 2. Experimental

All chemicals used were purchased from commercial sources and used without further purification. To a solution of 1-(2-methoxyphenyl)ethanone (1.5 g, 10 mmol) and 9-ethyl-9*H*-carbazole-3-carbaldehyde (2.23 g, 10 mmol) in MeOH (20 ml) was added 50% KOH (4 ml) aqueous solution dropwise with continuous stirring at room temperature. The reaction mixture was then refluxed for 4 h. The reaction suspension was poured onto cold H_2_O and the mixture was neutralized with 2 *M* HCl until the solution was acidic. A yellow solid was precipitated out, collected and washed with H_2_O. This solid was characterized by NMR to be the title compound. Crystals were grown from MeOH/H_2_O (50:1 *v*/*v*) solution by slow evaporation.

### 3. Refinement

For each methyl group, the hydrogen atoms were added in calculated positions using a riding-model with C—H = 0.98 Å and U(H) = 1.5*U*_eq_(C). The torsion angle, which defines the orientation of the methyl group about the C—C or O—C bond, was refined. The rest of the hydrogen atoms were included in calculated positions using a riding-model approximation with C—H = 0.95 to 0.99 Å and U_iso_(H) = 1.2*U*_eq_(C).

### Crystal data

**Table:**

C_24_H_21_NO_2_	*F*(000) = 1504
*M**_r_* = 355.42	*D*_x_ = 1.235 Mg m^−^^3^
Orthorhombic, *P**b**c**a*	Mo *K*α radiation, λ = 0.71073 Å
Hall symbol: -P 2ac 2ab	Cell parameters from 3814 reflections
*a* = 15.9332 (7) Å	θ = 1.0–25.0°
*b* = 7.8915 (3) Å	µ = 0.08 mm^−^^1^
*c* = 30.4062 (14) Å	*T* = 150 K
*V* = 3823.2 (3) Å^3^	Rectangular plate, pale yellow
*Z* = 8	0.35 × 0.27 × 0.02 mm

### Data collection

**Table:**

Nonius KappaCCD diffractometer	3352 independent reflections
Radiation source: Enraf Nonius FR590	1845 reflections with *I* > 2σ(*I*)
Horizonally mounted graphite crystal monochromator	*R*_int_ = 0.070
Detector resolution: 9 pixels mm^-1^	θ_max_ = 25.0°, θ_min_ = 1.3°
φ and ω scans	*h* = −18→18
Absorption correction: multi-scan (*HKL**SCALEPACK*; Otwinowski & Minor, 1997)	*k* = −9→9
*T*_min_ = 0.746, *T*_max_ = 0.998	*l* = −36→35
31757 measured reflections	

### Refinement

**Table:**

Refinement on *F*^2^	Primary atom site location: structure-invariant direct methods
Least-squares matrix: full	Secondary atom site location: difference Fourier map
*R*[*F*^2^ > 2σ(*F*^2^)] = 0.048	Hydrogen site location: inferred from neighbouring sites
*wR*(*F*^2^) = 0.102	H-atom parameters constrained
*S* = 1.00	*w* = 1/[σ^2^(*F*_o_^2^) + (0.0412*P*)^2^ + 0.295*P*] where *P* = (*F*_o_^2^ + 2*F*_c_^2^)/3
3352 reflections	(Δ/σ)_max_ < 0.001
246 parameters	Δρ_max_ = 0.16 e Å^−^^3^
0 restraints	Δρ_min_ = −0.14 e Å^−^^3^
0 constraints	

### Special details

**Table:**

Experimental. All work was done at 150 K using an Oxford Cryosystems Cryostream Cooler.The data collection strategy was set up to measure a quadrant of reciprocal space with a redundancy factor of 3.0, which means that 90% of the data was measured at least 3.0 times. Phi and omega scans with a frame width of 0.9 degree were used. Data integration was done with *DENZO*, and scaling and merging of the data was done with *SCALEPACK*.
Geometry. All e.s.d.'s (except the e.s.d. in the dihedral angle between two l.s. planes) are estimated using the full covariance matrix. The cell e.s.d.'s are taken into account individually in the estimation of e.s.d.'s in distances, angles and torsion angles; correlations between e.s.d.'s in cell parameters are only used when they are defined by crystal symmetry. An approximate (isotropic) treatment of cell e.s.d.'s is used for estimating e.s.d.'s involving l.s. planes.
Refinement. For each methyl group, the hydrogen atoms were added at calculated positions using a riding model with U(H) = 1.5 * *U*_eq_(bonded carbon atom). The torsion angle, which defines the orientation of the methyl group about the C—C or O—C bond, was refined. The rest of the hydrogen atoms were included in the model at calculated positions using a riding model with U(H) = 1.2 * *U*_eq_(bonded atom).

### Fractional atomic coordinates and isotropic or equivalent isotropic displacement parameters (Å^2^)

**Table:**

	*x*	*y*	*z*	*U*_iso_*/*U*_eq_	
C1	0.58107 (13)	0.4180 (3)	0.16865 (7)	0.0365 (5)	
C2	0.55972 (13)	0.4813 (3)	0.12727 (7)	0.0425 (6)	
H2	0.5137	0.4363	0.1112	0.051*	
C3	0.60792 (14)	0.6114 (3)	0.11058 (7)	0.0477 (6)	
H3	0.5943	0.6574	0.0826	0.057*	
C4	0.67642 (14)	0.6775 (3)	0.13385 (7)	0.0492 (6)	
H4	0.7089	0.7663	0.1214	0.059*	
C5	0.69696 (13)	0.6144 (3)	0.17475 (7)	0.0451 (6)	
H5	0.7433	0.6594	0.1906	0.054*	
C6	0.64896 (12)	0.4840 (3)	0.19261 (7)	0.0345 (5)	
C7	0.65009 (12)	0.3956 (3)	0.23405 (7)	0.0339 (5)	
C8	0.70128 (12)	0.4038 (3)	0.27101 (7)	0.0362 (5)	
H8	0.7475	0.4797	0.2714	0.043*	
C9	0.68545 (12)	0.3019 (3)	0.30752 (7)	0.0355 (5)	
C10	0.61672 (12)	0.1890 (3)	0.30591 (7)	0.0404 (6)	
H10	0.6051	0.1203	0.3308	0.048*	
C11	0.56615 (13)	0.1754 (3)	0.26952 (7)	0.0410 (6)	
H11	0.5207	0.0976	0.269	0.049*	
C12	0.58310 (12)	0.2786 (3)	0.23322 (7)	0.0350 (5)	
C13	0.47419 (13)	0.1832 (3)	0.17763 (7)	0.0499 (7)	
H13A	0.4331	0.2524	0.161	0.06*	
H13B	0.4447	0.1334	0.2032	0.06*	
C14	0.50550 (18)	0.0424 (3)	0.14849 (9)	0.0828 (9)	
H14A	0.5365	0.0907	0.1236	0.124*	
H14B	0.4577	−0.0232	0.1375	0.124*	
H14C	0.5427	−0.032	0.1654	0.124*	
C15	0.74185 (12)	0.3152 (3)	0.34485 (7)	0.0372 (5)	
H15	0.7867	0.3933	0.3413	0.045*	
C16	0.74025 (12)	0.2341 (3)	0.38357 (7)	0.0379 (5)	
H16	0.696	0.1573	0.39	0.045*	
C17	0.80689 (13)	0.2638 (3)	0.41605 (7)	0.0369 (5)	
C18	0.80572 (12)	0.1765 (3)	0.45984 (7)	0.0337 (5)	
C19	0.88251 (13)	0.1376 (3)	0.47869 (7)	0.0426 (6)	
H19	0.9326	0.1663	0.4634	0.051*	
C20	0.88829 (15)	0.0581 (3)	0.51919 (8)	0.0493 (6)	
H20	0.9416	0.0324	0.5315	0.059*	
C21	0.81594 (16)	0.0168 (3)	0.54127 (7)	0.0507 (6)	
H21	0.8193	−0.0387	0.5689	0.061*	
C22	0.73854 (15)	0.0549 (3)	0.52376 (7)	0.0468 (6)	
H22	0.6889	0.0257	0.5393	0.056*	
C23	0.73321 (13)	0.1361 (3)	0.48332 (7)	0.0371 (5)	
C24	0.58291 (13)	0.1302 (3)	0.48541 (8)	0.0627 (7)	
H24A	0.5824	0.0061	0.4864	0.094*	
H24B	0.5348	0.1704	0.4682	0.094*	
H24C	0.5794	0.1753	0.5154	0.094*	
N	0.54168 (10)	0.2928 (2)	0.19338 (6)	0.0396 (5)	
O1	0.86753 (9)	0.35552 (18)	0.40737 (5)	0.0474 (4)	
O2	0.65910 (8)	0.18701 (19)	0.46524 (5)	0.0484 (4)	

### Atomic displacement parameters (Å^2^)

**Table:**

	*U*^11^	*U*^22^	*U*^33^	*U*^12^	*U*^13^	*U*^23^
C1	0.0346 (12)	0.0392 (13)	0.0359 (13)	0.0007 (11)	0.0055 (11)	0.0002 (12)
C2	0.0404 (14)	0.0500 (15)	0.0371 (14)	0.0000 (12)	0.0011 (11)	−0.0038 (12)
C3	0.0535 (15)	0.0543 (16)	0.0352 (14)	0.0037 (14)	0.0009 (12)	0.0049 (13)
C4	0.0544 (15)	0.0485 (16)	0.0447 (15)	−0.0076 (13)	0.0043 (13)	0.0064 (13)
C5	0.0425 (14)	0.0499 (15)	0.0430 (15)	−0.0075 (12)	−0.0024 (12)	0.0011 (13)
C6	0.0329 (12)	0.0359 (13)	0.0346 (13)	0.0000 (11)	0.0021 (11)	−0.0020 (11)
C7	0.0335 (12)	0.0341 (13)	0.0340 (13)	0.0005 (11)	0.0010 (11)	−0.0009 (11)
C8	0.0314 (12)	0.0358 (13)	0.0413 (14)	−0.0022 (11)	0.0022 (11)	−0.0006 (12)
C9	0.0332 (12)	0.0354 (13)	0.0379 (14)	0.0015 (11)	0.0005 (10)	−0.0002 (11)
C10	0.0380 (13)	0.0439 (14)	0.0393 (14)	−0.0003 (12)	0.0020 (11)	0.0048 (12)
C11	0.0344 (12)	0.0417 (14)	0.0467 (15)	−0.0083 (11)	0.0011 (11)	0.0041 (12)
C12	0.0316 (12)	0.0381 (13)	0.0353 (13)	0.0015 (11)	0.0017 (10)	−0.0004 (11)
C13	0.0438 (14)	0.0623 (17)	0.0436 (15)	−0.0172 (13)	−0.0059 (11)	0.0042 (13)
C14	0.098 (2)	0.063 (2)	0.087 (2)	−0.0234 (17)	0.0054 (18)	−0.0195 (17)
C15	0.0338 (12)	0.0378 (13)	0.0399 (14)	−0.0011 (11)	0.0003 (11)	0.0002 (11)
C16	0.0325 (12)	0.0418 (14)	0.0392 (13)	−0.0034 (11)	0.0015 (11)	−0.0002 (12)
C17	0.0333 (12)	0.0384 (13)	0.0391 (14)	0.0014 (12)	0.0042 (11)	−0.0034 (11)
C18	0.0319 (12)	0.0344 (13)	0.0348 (13)	−0.0029 (11)	0.0014 (10)	−0.0041 (10)
C19	0.0391 (13)	0.0468 (15)	0.0419 (15)	−0.0024 (12)	−0.0010 (11)	−0.0014 (12)
C20	0.0488 (15)	0.0516 (16)	0.0477 (16)	0.0036 (13)	−0.0094 (13)	−0.0023 (13)
C21	0.0688 (18)	0.0469 (16)	0.0364 (14)	−0.0024 (15)	−0.0019 (14)	−0.0003 (12)
C22	0.0507 (16)	0.0477 (15)	0.0420 (15)	−0.0090 (13)	0.0101 (12)	−0.0023 (12)
C23	0.0375 (14)	0.0372 (13)	0.0366 (14)	0.0001 (11)	−0.0004 (11)	−0.0032 (11)
C24	0.0386 (14)	0.0740 (18)	0.0756 (18)	−0.0062 (14)	0.0152 (13)	−0.0085 (15)
N	0.0344 (10)	0.0462 (12)	0.0383 (11)	−0.0048 (10)	−0.0032 (9)	0.0036 (10)
O1	0.0388 (9)	0.0559 (10)	0.0476 (10)	−0.0099 (8)	0.0025 (7)	0.0022 (8)
O2	0.0336 (9)	0.0624 (11)	0.0493 (10)	−0.0022 (8)	0.0064 (7)	0.0010 (8)

### Geometric parameters (Å, º)

**Table:**

C1—N	1.391 (2)	C13—H13B	0.99
C1—C2	1.396 (3)	C14—H14A	0.98
C1—C6	1.404 (3)	C14—H14B	0.98
C2—C3	1.379 (3)	C14—H14C	0.98
C2—H2	0.95	C15—C16	1.340 (3)
C3—C4	1.402 (3)	C15—H15	0.95
C3—H3	0.95	C16—C17	1.469 (3)
C4—C5	1.379 (3)	C16—H16	0.95
C4—H4	0.95	C17—O1	1.236 (2)
C5—C6	1.393 (3)	C17—C18	1.499 (3)
C5—H5	0.95	C18—C19	1.386 (3)
C6—C7	1.440 (3)	C18—C23	1.395 (3)
C7—C8	1.390 (3)	C19—C20	1.385 (3)
C7—C12	1.411 (3)	C19—H19	0.95
C8—C9	1.394 (3)	C20—C21	1.373 (3)
C8—H8	0.95	C20—H20	0.95
C9—C10	1.412 (3)	C21—C22	1.377 (3)
C9—C15	1.452 (3)	C21—H21	0.95
C10—C11	1.373 (3)	C22—C23	1.389 (3)
C10—H10	0.95	C22—H22	0.95
C11—C12	1.398 (3)	C23—O2	1.363 (2)
C11—H11	0.95	C24—O2	1.432 (2)
C12—N	1.384 (2)	C24—H24A	0.98
C13—N	1.461 (2)	C24—H24B	0.98
C13—C14	1.506 (3)	C24—H24C	0.98
C13—H13A	0.99		
			
N—C1—C2	129.15 (19)	C13—C14—H14B	109.5
N—C1—C6	109.29 (18)	H14A—C14—H14B	109.5
C2—C1—C6	121.5 (2)	C13—C14—H14C	109.5
C3—C2—C1	117.5 (2)	H14A—C14—H14C	109.5
C3—C2—H2	121.2	H14B—C14—H14C	109.5
C1—C2—H2	121.2	C16—C15—C9	129.8 (2)
C2—C3—C4	121.7 (2)	C16—C15—H15	115.1
C2—C3—H3	119.2	C9—C15—H15	115.1
C4—C3—H3	119.2	C15—C16—C17	120.0 (2)
C5—C4—C3	120.4 (2)	C15—C16—H16	120
C5—C4—H4	119.8	C17—C16—H16	120
C3—C4—H4	119.8	O1—C17—C16	121.00 (19)
C4—C5—C6	119.2 (2)	O1—C17—C18	117.93 (18)
C4—C5—H5	120.4	C16—C17—C18	121.00 (19)
C6—C5—H5	120.4	C19—C18—C23	118.0 (2)
C5—C6—C1	119.7 (2)	C19—C18—C17	117.27 (19)
C5—C6—C7	133.8 (2)	C23—C18—C17	124.75 (19)
C1—C6—C7	106.50 (18)	C20—C19—C18	121.8 (2)
C8—C7—C12	119.25 (18)	C20—C19—H19	119.1
C8—C7—C6	133.78 (19)	C18—C19—H19	119.1
C12—C7—C6	106.96 (17)	C21—C20—C19	119.1 (2)
C7—C8—C9	120.71 (19)	C21—C20—H20	120.5
C7—C8—H8	119.6	C19—C20—H20	120.5
C9—C8—H8	119.6	C20—C21—C22	120.7 (2)
C8—C9—C10	118.47 (19)	C20—C21—H21	119.6
C8—C9—C15	117.97 (18)	C22—C21—H21	119.6
C10—C9—C15	123.55 (19)	C21—C22—C23	119.9 (2)
C11—C10—C9	122.2 (2)	C21—C22—H22	120.1
C11—C10—H10	118.9	C23—C22—H22	120.1
C9—C10—H10	118.9	O2—C23—C22	123.10 (19)
C10—C11—C12	118.51 (19)	O2—C23—C18	116.32 (18)
C10—C11—H11	120.7	C22—C23—C18	120.5 (2)
C12—C11—H11	120.7	O2—C24—H24A	109.5
N—C12—C11	130.25 (19)	O2—C24—H24B	109.5
N—C12—C7	108.86 (17)	H24A—C24—H24B	109.5
C11—C12—C7	120.86 (19)	O2—C24—H24C	109.5
N—C13—C14	112.70 (19)	H24A—C24—H24C	109.5
N—C13—H13A	109.1	H24B—C24—H24C	109.5
C14—C13—H13A	109.1	C12—N—C1	108.39 (16)
N—C13—H13B	109.1	C12—N—C13	126.16 (18)
C14—C13—H13B	109.1	C1—N—C13	125.12 (18)
H13A—C13—H13B	107.8	C23—O2—C24	117.99 (17)
C13—C14—H14A	109.5		
			
N—C1—C2—C3	177.6 (2)	C9—C15—C16—C17	177.50 (19)
C6—C1—C2—C3	0.1 (3)	C15—C16—C17—O1	−4.2 (3)
C1—C2—C3—C4	0.6 (3)	C15—C16—C17—C18	179.06 (18)
C2—C3—C4—C5	−0.7 (3)	O1—C17—C18—C19	−29.0 (3)
C3—C4—C5—C6	0.1 (3)	C16—C17—C18—C19	147.86 (19)
C4—C5—C6—C1	0.5 (3)	O1—C17—C18—C23	149.2 (2)
C4—C5—C6—C7	−176.7 (2)	C16—C17—C18—C23	−33.9 (3)
N—C1—C6—C5	−178.57 (18)	C23—C18—C19—C20	1.2 (3)
C2—C1—C6—C5	−0.6 (3)	C17—C18—C19—C20	179.58 (19)
N—C1—C6—C7	−0.6 (2)	C18—C19—C20—C21	−0.1 (3)
C2—C1—C6—C7	177.29 (18)	C19—C20—C21—C22	−0.6 (3)
C5—C6—C7—C8	−2.6 (4)	C20—C21—C22—C23	0.0 (3)
C1—C6—C7—C8	179.9 (2)	C21—C22—C23—O2	−175.86 (19)
C5—C6—C7—C12	178.0 (2)	C21—C22—C23—C18	1.3 (3)
C1—C6—C7—C12	0.5 (2)	C19—C18—C23—O2	175.48 (18)
C12—C7—C8—C9	−2.0 (3)	C17—C18—C23—O2	−2.7 (3)
C6—C7—C8—C9	178.7 (2)	C19—C18—C23—C22	−1.8 (3)
C7—C8—C9—C10	0.6 (3)	C17—C18—C23—C22	179.97 (19)
C7—C8—C9—C15	179.30 (18)	C11—C12—N—C1	177.8 (2)
C8—C9—C10—C11	0.9 (3)	C7—C12—N—C1	−0.2 (2)
C15—C9—C10—C11	−177.79 (19)	C11—C12—N—C13	−8.5 (3)
C9—C10—C11—C12	−0.8 (3)	C7—C12—N—C13	173.47 (18)
C10—C11—C12—N	−178.5 (2)	C2—C1—N—C12	−177.2 (2)
C10—C11—C12—C7	−0.7 (3)	C6—C1—N—C12	0.5 (2)
C8—C7—C12—N	−179.69 (17)	C2—C1—N—C13	9.1 (3)
C6—C7—C12—N	−0.2 (2)	C6—C1—N—C13	−173.23 (18)
C8—C7—C12—C11	2.1 (3)	C14—C13—N—C12	−95.3 (2)
C6—C7—C12—C11	−178.43 (18)	C14—C13—N—C1	77.3 (3)
C8—C9—C15—C16	179.2 (2)	C22—C23—O2—C24	−10.3 (3)
C10—C9—C15—C16	−2.1 (3)	C18—C23—O2—C24	172.48 (19)

### Hydrogen-bond geometry (Å, º)

**Table:**

*D*—H···*A*	*D*—H	H···*A*	*D*···*A*	*D*—H···*A*
C2—H2···O1^i^	0.95	2.48	3.387 (3)	160
C13—H13*A*···O1^i^	0.99	2.47	3.379 (3)	153

Symmetry code: (i) *x*−1/2, *y*, −*z*+1/2.
